# Supplementary material for: The interactome of intact mitochondria by cross-linking mass spectrometry provides evidence for coexisting respiratory supercomplexes
Source: Mol Cell Proteomics. 2017 Dec 8;17(2):216–32. doi: 10.1074/mcp.RA117.000470 (PMC5795388; doi:10.1074/mcp.RA117.000470)
Supplement: Supplemental Data [file supp_RA117.000470_133922_0_supp_23978_fzffwf.zip › spectra_annotation/mito_DR_spectra_annotation/41-1-3-1-16-1.pdf]

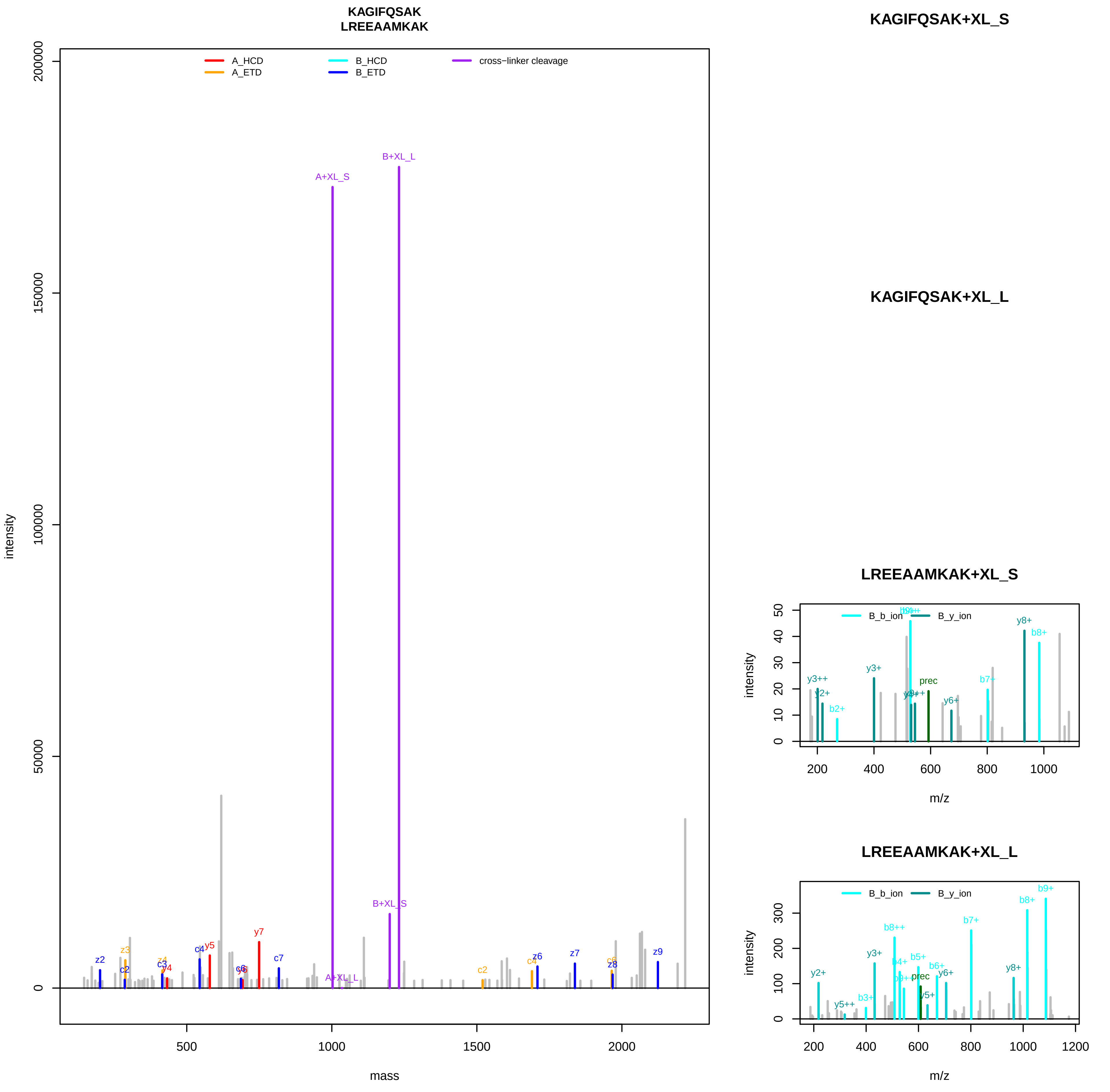

KAGIFQSAK
LREEAAMKAK
A_HCD
A_ETD
B_HCD
B_ETD
cross−linker cleavage
intensity
mass
A+XL_S
B+XL_L
B+XL_S
A+XL_L
z2
c2
z3
z4
c3
y4
c4
y5
c6
y6
y7
c7
c2
c4
z6
z7
c6
z8
z9
KAGIFQSAK+XL_S
KAGIFQSAK+XL_L
LREEAAMKAK+XL_S
B_b_ion
B_y_ion
y3++
y2+
b2+
y3+
y8+
b8+
b7+
y6+
prec
m/z
LREEAAMKAK+XL_L
b9+
b8+
b7+
b8++
y3+
y2+
b4+
b5+
b6+
y6+
prec
y5++
b3+
y5+
y8+
